# The spectrum of dermatological disorders among primary school children in Dar es Salaam

**DOI:** 10.1186/1471-2458-10-765

**Published:** 2010-12-16

**Authors:** Ewaldo V Komba, Yassin M Mgonda

**Affiliations:** 1 Department of Internal Medicine, Muhimbili University of Health and Allied Sciences, Dar es Salaam, Tanzania

## Abstract

**Background:**

Dermatologic disorders are common in many countries but the spectrum varies greatly. Many studies have reported a significant burden of skin diseases in school children. The objective of this study was to determine the current spectrum of dermatological disorders in primary school children in Dar es Salaam city.

**Methods:**

Primary school children were recruited by multistage sampling. Detailed interview, dermatological examination and appropriate laboratory investigations were performed. Data was analyzed using the 'Statistical Package for Social Sciences' (SPSS) program version 10.0 and EPI6. A p-value of < 0.5 was significant.

**Results:**

A total of 420 children were recruited (51% males; mean age 11.4 ± 2.8 years; range 6-19 years). The overall point prevalence of any skin disorder was 57.3% and it was 61.9% and 52.6% in males and females respectively (p = 0.05). Infectious dermatoses accounted for 30.4% with superficial fungal infections (dermatophytoses and pityriasis versicolor) being the commonest (20%). Dermatophytoses were diagnosed in 11.4% (48/420); the prevalence in males and females being 12.6% and 10.1% respectively (p = 0.41) and higher (21.8%) in the age-group 6-10 years (p = 0.045). Fungal cultures were positive in 42/48 children (88%). All three dermatophyte genera were isolated. Tinea capitis was the commonest disease among culture-positive dermatophytoses (30/42; 71.4%) with an overall prevalence of 7.1% (30/420) followed by tinea pedis (11/42; 26.1%) whose overall prevalence was 2.6%. *Microsporum canis *was common in tinea capitis (14/30; 46.7%) followed by *Trichophyton violaceum *(6/30; 20%). *Trichophyton rubrum *was common in tinea pedis (5/11; 45.5%). Thirty six children (8.6%) had pityriasis versicolor which was more prevalent (6/27; 22.l2%) in the age group 16-19 years (p = 0.0004). The other common infectious dermatoses were pyodermas (4%) and pediculosis capitis (3.6%). Common non-infectious dermatoses were: acne vulgaris (36.4%), non-specific dermatoses (10.7%), non-specific ulcers (5%) and atopic eczema (2.6%). Rare conditions (prevalence < 1%) included: vitiligo, alopecia areata and intertrigo. The majority of the affected children (67.2%) did not seek any medical assistance.

**Conclusions:**

Skin disorders are common in primary school children; infectious dermatoses are still rampant and many children do not seek medical assistance.

## Background

Studies from developing countries conducted over a period of years in the past have reported high prevalence of skin disorders among school children, the spectrum of which has been highly variable. In a review of prevalence studies in children by WHO, the prevalence of skin diseases ranging from 21% to 87% has been documented [1]. A study done in Ibadan, Nigeria more than a decade ago, reported the prevalence of skin disorders among school children of 35%. Infectious dermatoses were commonly observed, which included superficial fungal infections (tinea capitis and pityriasis versicolor) and scabies. Frequently observed non-infectious dermatoses were papular urticaria, angular cheilitis, melanocytic nevi, and tribal and therapeutic marks. Atopic eczema and viral warts were virtually absent [2]. Figueroa JI et al in a similar study in rural Ethiopia found a much higher prevalence (80%) of one or more skin diseases among school children. Infestations were the most prevalent followed by superficial fungal infections [3].

Elsewhere in the world, similar prevalence figures have been reported. In Turkey, Isil Inanir et al reported a prevalence of skin disorders of 77% among primary school children. Infectious dermatoses were frequently observed; pediculosis capitis, scabies, viral diseases and fungal infections [4].

A study done in rural Tanzania reported 55% of the school children as having one or more skin diseases [5]. Two more surveys in different rural Tanzanian communities among children and adults documented the prevalence of skin disorders of 35% and 27% respectively with infectious dermatoses comprising the bulk [6,7]. A more recent study from Iraq reported the overall prevalence of skin diseases among primary-school children of 40.9% and that this high prevalence may reflect the prevailing low socio-economic conditions [8]. Our study aimed at describing the current spectrum of dermatological disorders among urban primary school children in a city of a sub Saharan African country as compared to previous reports.

## Methods

### Study design and setting

This was a descriptive cross-sectional study conducted in public primary schools of Dar es Salaam city.

### Sampling technique

A multi stage sampling technique was employed. Primary schools were selected from all municipals of Dar es Salaam city using random number tables. A sampling fraction of 4.6% was used in each school to get the required number of children who were then recruited by a simple random technique. School children were recruited regardless of the strict age-definition of the term 'child,' provided they were still in primary schools.

### Clinical and laboratory work-up

Recruited children were interviewed and examined in day light. Dermatological diagnosis was made mainly clinically. Laboratory investigations were used to confirm difficult diagnoses. Skin scrapings, nail clippings and hairs were obtained as appropriate and treated for 15-30 minutes with 1-2 drops of 20% KOH before being examined microscopically for fungal hyphae. Fungal cultures were done on Sabouraud's Dextrose Agar and considered positive if there was growth on day 14 or 28 and negative if there was no growth on day 28. Pus swabs were collected for gram stain and cultures were done on Blood and MacConkey Agar. The bacterial cultures were considered negative if there was no growth after 48 hours of incubation. Specimens were obtained from burrows of suspected scabies lesions for direct microcopy for mites. Skin biopsies were taken as appropriate for routine histological examination. There were no special tests done to confirm viral lesions.

### Data analysis

Data was entered into a computer and counterchecked to ensure correctness of entries. Analysis was done using the Statistical Package for Social Sciences (SPSS) version 10.0. Chi-squared and Fisher's exact statistical tests were used to determine associations for categorical variables. A p-value of less than 0.05 was considered statistically significant.

### Ethical issues

Ethical clearance was obtained from the 'Muhimbili University of Health and Allied Sciences' Ethics Board. Confidentiality on candidate's information was maintained.

## Results

A total of 420 primary school children were recruited. Of these, 213 (51%) were males. The age ranged from 6 to 19 years, (mean 11.4 ± 2.8 years). Twenty seven pupils (6.4%) were much older, aged above 15 years. The mean family size was 6.3 ± 2.4 and the mean number of persons per room was 2.4 ± 1.1. The overall point prevalence of any skin disease was 57.3% (241/420) with 48% having one, 9% two and 1% three. The prevalence of any skin disorder was 61.9% (132/213) in males and 52.6% (109/207) in females (p = 0.05).

Table 1 shows the prevalence of skin diseases and their distribution by sex and age-groups. Infective dermatoses accounted for nearly one-third (128/420; 30.4%) of all skin disorders, with superficial fungal infections (dermatophytoses and pityriasis versicolor) accounting for 20% (84/420). Dermatophytoses diagnosed by KOH preparation were found in 48/420 children (11.4%) and the prevalence in males and females was 12.6% and 10.1%) respectively (p = 0.41). Furthermore, dermatophytoses were more prevalent (21.8%) among pupils aged 6-10 years (p = 0.045).

**Table 1:** Age and Sex distribution of primary school children with skin disorders in Dar es Salaam (n = 420)

Skin disorders	Sex			Age-group (years)				
	**M = 213**	**F** **= 207**	**P-value**	**6-10** **= 156**	**11-15** **= 237**	**16-19** **= 27**	**TOTAL**	**P-value**
	**n(%)**	**n(%)**		**n(%)**	**n(%)**	**n(%)**		

**Infective skin disorders**								
Dermatophytosis	27 (12.6)	21 (10.1)	0.41	23 (21.8)	24 (15.6)	1 (3.7)	**48** **(11.4)**	0.045
Pityriasis versicolor	23 (10.7)	13 (6.2)	0.09	4 (2.5)	26 (10.9)	6 (22.2)	**36** **(8.6)**	0.0004
Pyodermas	9 (4.2)	8 (3.9)	0.85	6 (3.8)	10 (4.2)	1 (3.7)	**17** **(4.0)**	-
Pediculosis capitis	5 (2.3)	10 (4.8)	0.17	7 (4.5)	8 (3.4)	0	**15** **(3.6)**	-
Scabies	4 (1.8)	2 (0.9)	0.70	1 (0.6)	5 (2.1)	0	**6** **(1.4)**	-
Viral warts	2 (0.9)	4 (1.9)	0.65	4 (2.5)	2 (0.8)	0	**6** **(1.4)**	-
**Non infective skin disorders**								
Acne vulgaris	78 (36.6)	75 (36.2)	0.93	0	143 (60.3)	10 (37.0)	**153** **(36.4)**	-
Non specific ulcers	14 (6.5)	7 (3.4)	0.133	10 (6.4)	11 (5.2)	0	**21** **(5)**	-
Atopic eczema	6 (2.8)	5 (2.4)	0.79	6 (3.8)	4 (1.7)	1 (3.7)	**11** **(2.6)**	-
Birth marks	6 (2.8)	4 (1.9)	0.78	6 (3.8)	3 (1.2)	1 (3.7)	**10** **(2.3)**	-
Seborrheic eczema	3 (1.4)	2 (0.9)	0.97	1 (0.6)	2 (0.8)	2 (7.4)	**5** **(1.2)**	-
Keratosis pilaris	5 (2.3)	0	-	3 (1.9)	2 (0.8)	0	**5** **(1.2)**	-
Angular cheilitis	2 (0.9)	3 (1.4)	0.97	3 (0.6)	2 (0.8)	0	**5** **(1.2)**	-
Vitiligo	3 (1.4)	0	-	1 (0.6)	2 (0.9)	0	**3** **(0.7)**	-
Alopecia areata	1 (0.4)	0	-	0	1 (0.4)	0	**1** **(0.2)**	-
Intertrigo	1 (0.4)	0	-	0	1	0	**1** **(0.2)**	0.000
Non specific dermatoses	29 (13.6)	16 (7.7)	0.05	8 (5.1)	12 (5.0)	25 (92.2)	**45** **(10.7)**	0.0001
**Any skin disorder**	132 (61.9)	109 (52.6)	0.05	71 (45.5)	148 (62.4)	22 (81.5)	**241** **(57.3)**	

**NB: **p-values could not be calculated for conditions with very small numbers

Fungal cultures were performed for all 48 children and were positive in 42/48 (88%). These results have been presented separately in table 2. All three dermatophyte genera were isolated: *Trichophyton *(24/42; 57.1%), *Microsporum *(17/42; 40.4%) and *Epidermophyton *(1/42; 2.4%). The most prevalent culture-positive dermatophytosis was tinea capitis observed in 71.4% (30/42); giving an overall prevalence of 7.1% (30/420) and *M. canis *was the commonest species isolated in 46.7% (14/30), followed by *T. violaceum *(20%). T. pedis was encountered in 26.1% (11/42), giving an overall prevalence of 2.6% (11/420) and *T. rubrum *was the leading etiology present in 45.4% (5/11) followed by *T. mentagropyhtes *in 36.4% (4/11). *E. floccosum *and *T. schoenleinii *were also isolated from children with tinea pedis; each in one child (9.1%). Only one of 42 culture-positive children (2.3%) had tinea corporis due to *M. canis*, giving an overall prevalence of 0.2% (1/420).

**Table 2:** The distribution of isolated dermatophytes according to the type of ringworm

	Type of ringworm			
		
Dermatophytes	Tinea capitis n (%)	Tinea pedis n (%)	Tinea corporis n (%)	Total n (%)
*E. floccosum*	0 (0.0)	1 (9.1)	0 (0.0)	1 (2.4)
*M. audouinii*	2 (6.7)	0 (0.0)	0 (0.0)	2 (4.8)
*M. canis*	14 (46.7)	0 (0.0)	1 (100.0)	15 (35.7)
*T. mentagrophytes*	4 (13.3)	4 (36.4)	0 (0.0)	8 (19.0)
*T. rubrum*	3 (10.0)	5 (45.5)	0 (0.0)	8 (19.0)
*T. schoenleinii*	1 (3.3)	1 (9.1)	0 (0.0)	2 (4.8)
*T. violaceum*	6 (20.0)	0 (0.0)	0 (0.0)	6 (14.3)
Total	30 (100.0)	11 (100.0)	1 (100.0)	42

Apart from dermatophytoses, the other superficial fungal infection encountered was pityriasis versicolor in 36 children (8.6%), which was more prevalent (6/27; 22.l2%) in the older age group; 16-19 years (p = 0.0004). Other relatively common infective dermatoses included pyodermas (4%) and pediculosis capitis (3.6%). Scabies and viral warts were less frequent (prevalence 1.4%). Of the pyodermas, 13 (3.0%) were folliculitis while one child had carbuncle and only 6 specimens grew bacteria: *Staphylococcus aureus *(3), *Klebsiella pneumonia *(2) and *Escherichia coli *(1).

There was a wide range of non-infective skin disorders. Many of these conditions, however, were of very low prevalence. Overall, acne vulgaris was the commonest, being encountered in 36.4% and more prevalent in children of age-group 11-15 years, (60.3%; p = 0.000). Twenty one children (5%) had mild, superficial non-specific ulcers. Atopic eczema and birthmarks had prevalence of 2.6% and 2.3% respectively. Seborrheic eczema, keratosis pilaris and angular cheilitis were less frequent, each with prevalence of 1.2%. Conditions with very low prevalence (< 1%) were vitiligo, alopecia areata, and intertrigo. Non-specific dermatoses occurred in 10.7% (45/420), being common (25/27; 92.2%) in older children aged 16-19 years (p = 0.0001).

The majority of the children (67.2%) did not seek any medical care for their skin diseases. Few (16.6%), visited hospitals while others (14.5%) practiced self medication and a minority went to traditional healers (1.2%) or used Chinese medicine (0.4%).

## Discussion

This study has documented the current spectrum of skin diseases among school children in an urban setting of a developing sub Saharan African country. The point prevalence of any skin disorder of 57% is alarmingly high and still comparable to that of a previous study (55%) among school children in rural Tanzania [5]. Other previous studies from developing countries have reported almost similar figures varying between 35% and 80% [2,3]. The total skin disease burden is most likely underestimated in our study since point prevalence studies often miss diseases of short duration.

Many studies have reported infectious dermatoses as comprising more than one-third of all skin disorders [2,3,5,7,9]. In our study, infectious dermatoses similarly comprised almost one-third (30.4%), and dermatophyte infections were the most frequent (11.4%) with culture-proven tinea capitis having the highest overall prevalence (7.1%). A Nigerian study [2] conducted more than a decade ago reported the prevalence of tinea capitis, which was higher (15%) than ours, but our study considered only culture-positive diseases. *Microsporum canis *was the commonest isolate (35.7%), followed by *Trichophyton mentagrophytes *(19%) and *Trichophyton rubrum *(19%). In an Iranian study conducted one and a half decades ago, Khosravi AR et al, also found the highest frequency with *Microsporum canis *(19%) followed by *Trichophyton rubrum *(16.5% and *Epidermophyton floccosum *(15%) [10]. Elsewhere in the world studies have reported different species [11-13]. In our study one child had tinea capitis caused by *T. rubrum*, while another one had tinea pedis caused by *E. floccosum*, and yet another one had tinea pedis due to *T. schoenleinii*. All these are rare findings. Although the numbers involved are very small, these findings call for more comprehensive studies to define clearly the prevalence of these organisms and their role in causing different fungal diseases in African primary school children. Our study also found tinea pedis to be more prevalent (2.6%) than tinea corporis (0.2%) which is not usual in children. This could be due to the fact that our study was conducted in Dar es Salaam, a city with a hot, humid climate almost throughout the year, with school children having adopted the western style of life, of wearing closed shoes at all times while at school and most of the times at homes all of which would make them at risk of developing tinea pedis. Tinea corporis on the other hand is usually more common in rural areas.

The older children were more likely to have pityriasis versicolor as the condition is more common in the post-pubertal age where sebaceous glands are active [14]. The lower prevalence of scabies in this study (1.4%) is notable but not exceptional. Other studies conducted in preschool children, rural community, households and under-five children in refugee camps have recorded wide prevalence variations between 0.4% and 77% [15-17]. Differences in socioeconomic standards, even within countries have been mentioned as some of the factors responsible for such great variations in prevalence [2,17,8].

Atopic eczema and seborrheic eczema were observed with lower frequencies of 2.6% and 1.2% respectively. This is consistent with observations in the general population where atopic eczema and seborrheic eczema in children have been found to vary from less than 1% to 5% [1]. Furthermore, Ogunbiy OA et al did not find any cases of atopic eczema among the school children they studied [2].

The majority of the school children in this study (67%) did not seek any form of treatment for their skin conditions. The low level of medical care seeking behavior has also been demonstrated among secondary school children with acne vulgaris [18]. This attitude may be attributable to the assumption that skin diseases are a benign minor nuisance, not meriting any treatment [1].

Unlike non-infective dermatoses, many of which are genetic, autoimmune or idiopathic in etiology and therefore difficult to prevent, many infectious dermatoses are easy to treat and even prevent. It is surprising therefore to find a trend of infectious skin diseases which is still similar to that described several decades ago.

## Conclusions

Skin disorders are common among school children affecting about two thirds of them but the majority does not seek medical advice. There is need to strengthen school health programs so that regular skin examination and proper advice to affected children can be provided.

## Study limitations

This being a point prevalence cross-section study, the total skin disease burden is most likely underestimated since point prevalence studies often miss diseases of short duration.

## Competing interests

The authors declare that they had no competing interests during the design up to the conduct of this study.

## Authors' contributions

EVK participated in the study design and conducted data collection. YMM conceived the study theme, participated in the study design, supervised data collection and prepared the final manuscript. Both authors read and approved the final manuscript.

## Pre-publication history

The pre-publication history for this paper can be accessed here:

http://www.biomedcentral.com/1471-2458/10/765/prepub
